# Using process mapping to identify barriers to effective management of sepsis in a cancer hospital: lessons for successful implementation of a whole hospital pathway

**DOI:** 10.1186/cc14056

**Published:** 2014-12-03

**Authors:** K Thursky, G Haeusler, B Teh, D Comodo, N Dean, C Brown, J Willis, K Morris, S Lingaratnam, M Slavin, L Worth

**Affiliations:** 1 Department of Infectious Diseases, Peter MacCallum Cancer Centre, Melbourne, Australia; 2 Nursing, Peter MacCallum Cancer Centre, Melbourne, Australia; 3 Pharmacy Department, Peter MacCallum Cancer Centre, Melbourne, Australia

## Introduction

Infection and sepsis are common problems in cancer management affecting up to 45% of patients. However, international guidelines focus on the management of neutropenic fever, and fail to address the recognition and resuscitation of patients who meet sepsis criteria. Peter MacCallum Hospital is a 100 inpatient-bed tertiary cancer hospital with hematology, medical oncology, cancer surgery and radiation oncology, as well as a medical and chemotherapy day unit, apheresis, and large ambulatory service but no emergency department. Records showed that up to 25% of all in-hospital Medical Emergency Team (MET) calls were attributable to sepsis with in-hospital mortality rates of up to 25%. We aimed to identify barriers to effective management of inpatient sepsis at Peter MacCallum Cancer Centre and to implement a hospital-wide sepsis pathway.

## Methods

**Figure 1:**
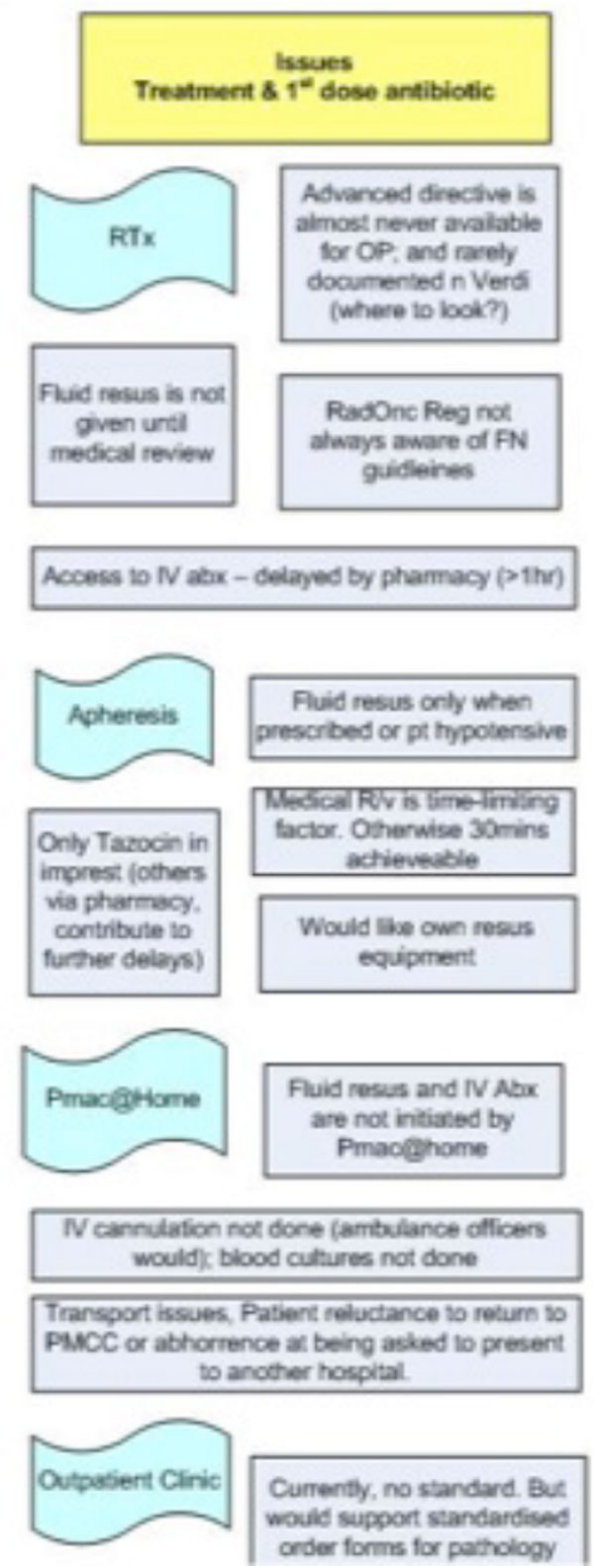
**Portion of process map identifying barriers to timely administration of antibiotics in the ambulatory setting**. RTx, radiotherapy department.

A sepsis working party was formed with the antimicrobial stewardship team, clinicians, and senior ambulatory and inpatient nurses. Each member undertook direct observation and focus group interviews in an allocated clinical area. Three key areas were examined: issues relating the identification of sepsis, issues relating to clinical review of the patient, and issues relating to timely administration of first dose of antibiotic. Inpatient and outpatient issues were graphically represented in a process map (Figure 1).

## Results

**Figure 2:**
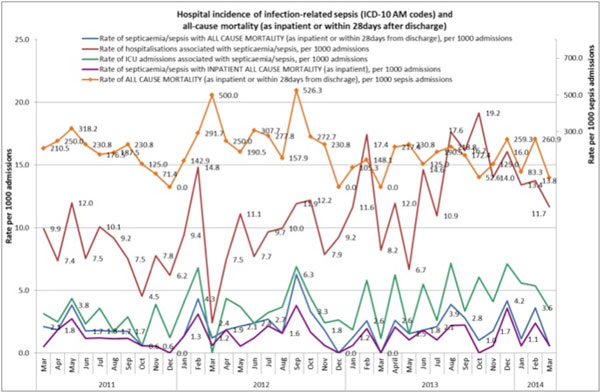
**Increased rate of sepsis at Peter MacCallum Cancer Centre from March 2013, coinciding with improved identification of patients with sepsis**.

Process mapping revealed significant gaps in knowledge in medical and nursing staff and structural barriers to rapid resuscitation of patients. There were significant knowledge gaps in the awareness of sepsis diagnostic criteria, the role of lactate, effective fluid resuscitation, and the need for early clinical review and referral to the ICU. Examples of structural barriers to effective resuscitation included lack of availability of nurse cannulators, availability of antibiotics, rapid intravenous fluid infusers and sufficient after-hours medical and ICU liaison support. Knowledge and structural barriers were systematically addressed during the implementation of the clinical sepsis pathway. The sepsis pathway was designed as a medical record form to be used across all clinical areas, and supports nurse initiation. Following pathway implementation in March 2013 there have been substantial improvements in the number of patients who have had a lactate taken, received appropriate fluid resuscitation and time to first dose of antibiotics. Administrative data shown in Figure 2 demonstrate increased ascertainment of cases, and a fall in all-cause sepsis mortality after the pathway was commenced.

## Conclusion

Identifying knowledge gaps and structural barriers using process mapping led to the successful design and implementation of a sepsis program. The figures show that despite increased rates of coded sepsis cases, mortality and ICU admission rates have not increased. Mortality of patients coded for sepsis March to October 2012 (13/56 (23.2%)) compared with those for March to October 2013 (10/121 (8.3%)).

